# Virtual Dual-Loop Feedback Control with Model-Construction Linear Extended State Observer for Free Space Optical Communication

**DOI:** 10.3390/s19183846

**Published:** 2019-09-06

**Authors:** Kang Nie, Wei Ren, Xi Zhou, Yao Mao

**Affiliations:** 1 Key Laboratory of Optical Engineering, Chinese Academy of Sciences, Chengdu 610209, China (K.N.) (W.R.) (X.Z.); 2 Institute of Optics and Electronics, Chinese Academy of Sciences, Chengdu 610209, China; 3 University of Chinese Academy of Sciences, Beijing 100049, China

**Keywords:** free space optical communication, optoelectronic target detector, model-construction linear extended state observer, virtual dual-loop feedback control, tip-tilt mirror, disturbance suppression

## Abstract

The stable alignment and transmission of free space optical communication (FSO) is susceptible to internal dynamics and external disturbances. In this paper, a virtual dual-loop feedback control (VDFC) with model-construction linear extended state observer (MCLESO), which is applied to the fast tip-tilt mirror platform to enhance the disturbance suppression ability (DSA) for FSO. MCLESO, which is modified on a classical linear extended state observer by introducing the available model information, is shown to use the input and output signal data of the system to observe total disturbances, including internal dynamics and external disturbance. Since the position and velocity signals are both observed only with the optoelectronic target detector and MCLESO, the controllers of the dual-loop feedback control (DFC) system are employed directly. This method has a more accurate control performance after model construction, which enhances the DSA of the tip-tilt mirror control system in low and medium frequency. It is also beneficial to miniaturization and cost saving by not using velocity sensors. Both simulations and experiments validate the effectiveness of the proposed method in the tip-tilt mirror control system under the condition of disturbance.

## 1. Introduction

Free-space optical communication has the advantages of high communication rate, small size, low power consumption and strong confidentiality, and has a very broad application prospect. Due to the narrow laser beam and small divergence angle, the construction of large-capacity, long-distance, and highly reliable optical communication links relies on the continuous alignment between the two terminals of optical communication. The acquisition, tracking and pointing (ATP) is still the key technology for FSO [1,2,3,4]. The two-dimensional fine pointing tip-tilt mirror is used as a control component to reflect the beacon of light into the optoelectronic target detector, and the light position is measured by a high frame rate detector. The spot is centered by position closed-loop control [5,6]. The final stabilization precision of tip-tilt mirror is the guarantee to establish optical communication links and maintain stable transmission.

However, tip-tilt mirror is susceptible to internal dynamics and external disturbances. The internal dynamics include changes in the structure and parameters of the controlled object affected by the environment, pose and load. External disturbances include wind disturbances and vibrations of the base carrier, mostly concentrated in 100 Hz [7,8]. These will cause the spot to produce offset and jitter on target detector, in order to overcome the interference during communication, and maintain the optical path stability. In the case that the platform’s own hardware conditions cannot be changed, using high-performance control algorithms to enhance the active disturbance rejection capability of the tip-tilt mirror is particularly important.

A direct feedback loop using position error from high-resolution Position Sensitive Detector (PSD) is utilized to control light in deep space communication by the Draper Laboratory [9,10]. High sample rate inertial sensors, such as fiber optic gyroscopes (FOGs) and the micro-electro-mechanical system (MEMS) accelerometers, have been utilized to compose the acceleration loop, velocity loop and position loop of the multi-loop feedback control (MFC) system, its total disturbance suppression ability (DSA) is the product of the effects of each loop [11,12], but these methods require the installation of additional inertial sensors on the tip-tilt mirror platform, which is detrimental to its small inertia and rapidity, as well as increases space and economic costs. The direct feedforward method based on measurement is recommended to suppress the external vibrations which are measured by sensors on the base, but this requires accurate identification of the disturbance transfer characteristics from the base to the tip-tilt mirror [13,14]. Ohnishi proposed a feedforward compensation control technique based on disturbance observer (DOB) in 1996. The estimated external disturbance is observed indirectly and compensated [15,16]. Tang introduced the DOB into the charge-coupled device (CCD)-based control loop to enhance the disturbance rejection capability [17]. However, the characteristics of the plant model cannot be accurately identified and the ideal compensators are often non-causal. DSA is limited by system stability and filter design [18]. Various advanced control techniques have been proposed recently such as the sliding model control, $H_{2}/H_{\infty }$ synthesis control, learning control and so on [19,20,21], but the dependence on the precise model and the complexity of the controller are difficult in engineering practices. To overcome undesired changes that have an impact on the system output without a high-precision mathematical model [22], Han proposed active disturbance rejection control (ADRC) which is a nonlinear control algorithm to deal with unknown dynamics and disturbance. The basic idea of ADRC is to use an extended state observer (ESO) to estimate the “total disturbances”, and this makes the feedback control more robust and less dependent on the detailed mathematical model of the physical process. Then, through disturbance compensation, the originally complex and uncertain plant dynamics are reduced to a simple cascade integral plant [23]. However, the tuning procedure of ADRC is very complicated due to its large number of parameters, and theoretical analysis is also difficult. Gao simplified ADRC to linear ADRC (LADRC) using linear ESO (LESO) and linear controller in place of the original nonlinear parts. Simply, workers only need to tune two parameters: observer bandwidth and controller bandwidth, which can be adjusted by the frequency domain target of the controlled system [24,25]. Thus, this made the tuning of linear ADRC (LADRC) more realistic. Studies showed that the LADRC still achieved high performance and good robustness through theoretical proofs in the frequency and time domains [26,27,28].

The approximate model information of the tip-tilt mirror can be identified by physical modeling or frequency response analysis, and the traditional closed-loop controller is designed according to this known model. Therefore, researchers added available model information in the design of LESO to reduce the observer burden [29,30]. Same as LADRC, the known model dynamics are also included in the total disturbance that needs to be compensated, and the controller bandwidth also needs to be adjusted to a PD controller. In order to retain the original closed-loop controller and make full use of the available model information, in this paper, a MCLESO is proposed to observe its own states and unknown disturbance. The known dynamics are not included in the total disturbance to be compensated. The uncertain plant is constructed to available model dynamics by compensating the unknown internal dynamics and external disturbance. Then, the position and velocity states observed by MCLESO are composed to implement VDFC. The proposed strategy can directly use the traditional DFC controller and save extra space and cost of the velocity sensors. The VDFC with MCLESO applied in the fast tip-tilt mirror platform enhances the DSA in low and medium frequency, compared with traditional DFC and LADRC in simulations and experiments.

The remainder of the paper is organized as follows. Section 2 presents an introduction to the tip-tilt mirror control system. Section 3 describes MCLESO, its stability analysis and dynamic construction. Section 4 discusses the controller designs. Section 5 introduces the experimental platform and set up experiments to testify the effectiveness of the proposed method. Concluding remarks are presented in Section 6.

## 2. Tip-Tilt Mirror Control System

**Figure 1:**
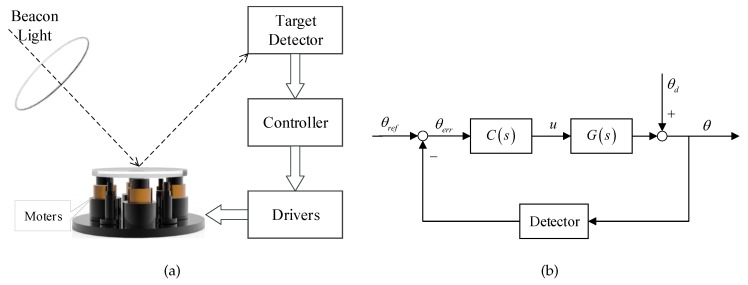
The schematic of the tip-tilt mirror control system: (**a**) structure diagram. (**b**) block diagram.

The tip-tilt mirror control system in FSO is mainly composed of a fast fine pointing tip-tilt mirror, high frame rate optoelectronic target detector, controller and driver modules. Its schematic structure is shown in Figure 1a. A detector such as PSD receives the beacon of light reflected by the tip-tilt mirror, and sends the position error signal to the controller. The controller calculates the correction angle of the mirror, and then through D/A converter, the output of controller drives the motors connected to the mirror. The aim is to stabilize the light at the center of the detector by rapidly deflecting the mirror under the influence of the disturbance. Figure 1b is the classical feedback control block diagram. *G* is the controlled mirror platform, *C* is the position controller, $\theta_{ref}$ is the given target position, θ is the position output, *u* is the input of the controlled plant, and $\theta_{d}$ is the outer disturbance angle.

**Figure 2:**
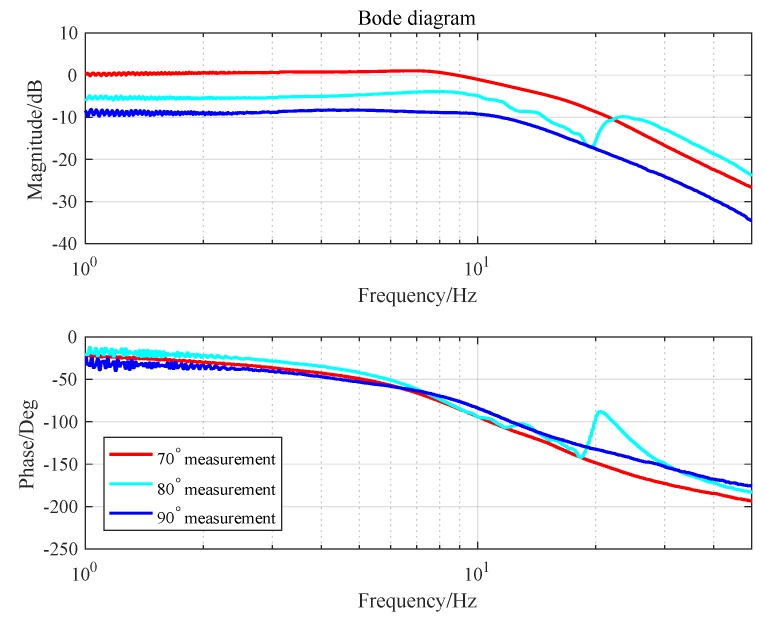
The open-loop frequency characteristics of the plant in different attitudes.

For the tip-tilt mirror, the frequency characteristics from the voltage input to the angle output can be approximated to a typical resonance element. Therefore, the general form of the second-order controlled model in low and medium frequency can be expressed as follows.

(1) $$\begin{array}{c} G\left(s\right)=\frac{\theta (s)}{U(s)}=\frac{K}{\frac{s^{2}}{{\omega_{n}}^{2}}+\frac{2\zeta }{\omega_{n}}s+1}=\frac{b}{s^{2}+a_{1}s+a_{0}} \end{array}$$

where, $a_{1}=2\zeta \omega_{n}$, $a_{0}=\omega_{n}^{2}$, $b=K\omega_{n}^{2}$. By analyzing and fitting frequency response, the parameters of the controlled plant ($K,\omega_{n},\zeta$) are the plant gain, resonant frequency and damping ratio, respectively, and the identification of these parameters is usually not accurate enough, just some approximations. In an actual working environment, the tip-tilt mirror platform is not only affected by external disturbance, its own characteristics will also change as attitude and load change. Adjusting the attitude angle of the platform and measuring its open-loop frequency characteristics, the platform gain and resonant frequency will change accordingly. The measurement results are shown in Figure 2. If the controller is designed based on the parameters obtained from the original identification, the zeros and poles of the changed plant are hard to eliminate. Hence, the stability margin will be greatly affected.

## 3. Model-Construction Linear Extended State Observer (MCLESO)

### 3.1. Observer Design

**Figure 3:**
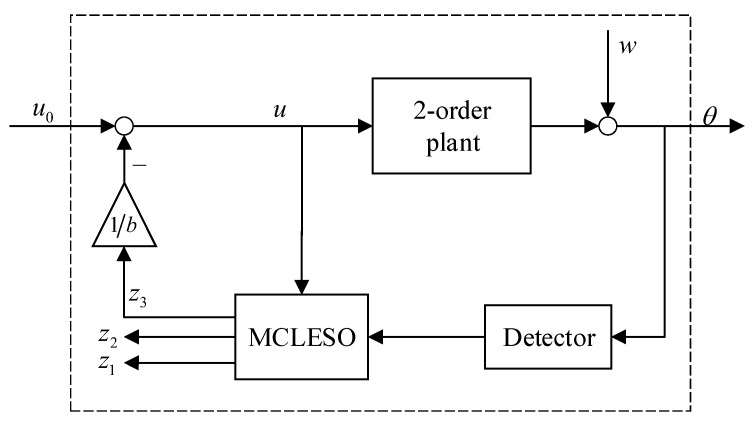
The block diagram of MCLESO for second-order plant.

Using a second-order plant as a research object in this section, the corresponding MCLESO observes the self states and the unknown disturbance of the controlled plant. Moreover, the unknown plant dynamics containing internal and external disturbances are constructed to available object dynamics. The block diagram of MCLESO observation and compensation for a second-order plant is shown in Figure 3.

A second-order controlled object such as (1) can be expressed as a differential equation

(2) $$\begin{array}{c} \ddot{\theta }=f\left(\theta ,\dot{\theta },w,t\right)+bu=f_{0}+f_{x}+f_{w}+bu \end{array}$$

where, $f_{0}=-a_{1}\dot{\theta }-a_{0}\theta$ is the partial model information obtained by identification, but $a_{0}$ and $a_{1}$ are usually not accurate enough, so $f_{x}$ represents the inaccurate part of the modeling and the changed part of the internal dynamics. $f_{w}$ is referred as the external disturbance. So $f\left(\theta ,\dot{\theta },w,t\right)=f=f_{0}+f_{x}+f_{w}$ is regarded as the combined effect of the known model dynamics and unknown disturbance, but traditional LADRC is independent of known information and define the whole *f* as a new augmented state observed by LESO. $f_{0}$ has high frequency response characteristics, the burden of observation will be greatly increased if the known model dynamics are not used, and in order to retain the original controllers design and achieve better control performance. We select the deflection angle and the angular velocity as two original states of the system, and $f^{\prime }=f_{x}+f_{w}$ as an augmented state. Then, the state variables of the extended system are as follows.

(3) $$\begin{array}{c} x={\left[\begin{array}{ccc} x_{1} & x_{2} & x_{3} \end{array}\right]}^{T}={\left[\begin{array}{ccc} \theta & \dot{\theta } & f^{\prime } \end{array}\right]}^{T} \end{array}$$

where ${\left[\right]}^{T}$ denotes transpose.

The plant in Equation (2) is written in state equation form

(4) $$\begin{array}{c} \left\{\begin{array}{cc} {\dot{x}}_{1} & =x_{2}\\ {\dot{x}}_{2} & =-a_{1}\dot{\theta }-a_{0}\theta +bu\\ {\dot{x}}_{3} & =\dot{f}{}^{\prime }\\ \theta & =x_{1} \end{array}\right. \end{array}$$

We can see that $f_{0}$ is included in the original state, not in the extended state $x_{3}$. The augmented state space form of Equation (4) is

(5) $$\begin{array}{c} \begin{array}{cc} \dot{x} & =Ax+Bu+E\dot{f}{}^{\prime }\\ y & =Cx \end{array} \end{array}$$

with

(6) $$\begin{array}{c} A=\left[\begin{array}{ccc} 0 & 1 & 0\\ -a_{0} & -a_{1} & 1\\ 0 & 0 & 0 \end{array}\right],B=\left[\begin{array}{c} 0\\ b\\ 0 \end{array}\right],C=\left[\begin{array}{ccc} 1 & 0 & 0 \end{array}\right],E=\left[\begin{array}{c} 0\\ 0\\ 1 \end{array}\right] \end{array}$$

Applying the design of state observer in linear system theory and imitate the LESO in Reference [24], the state space observer, denoted as the MCLESO, is

(7) $$\begin{array}{c} \begin{array}{cc} \dot{z} & =\left[A-LC\right]z+\left[B,L\right]u_{l}\\ y_{l} & =z \end{array} \end{array}$$

where, *z* is the state vector of the observer, which observes the state vector of Equation (5), that is $z_{i}\approx x_{i}\left(i=1,2,3\right)$. The inputs of observer are control signal *u* and plant output θ, that is $u_{l}={\left[\begin{array}{cc} u & y \end{array}\right]}^{T}$. The outputs $y_{l}$ of observer are *z*. The value of (A,B,C) are shown in Equation (6). L is the observer gain vector, which can be obtained using any known method such as the pole placement technique,

(8) $$\begin{array}{c} L={\left[\begin{array}{ccc} l_{1} & l_{2} & l_{3} \end{array}\right]}^{T} \end{array}$$

### 3.2. Stability Analysis

Let state error vector be e=x−z, and subtract Equation (7) from Equation (5). The matrix equation of observation error can be written as

(9) $$\begin{array}{c} e^{\prime }=\left(A-LC\right)e+Ef^{\prime } \end{array}$$

The MCLESO is bounded-input bounded-output (BIBO) stable if the $\left(A-LC\right)$ is Hurwitz and $f^{\prime }$ is bounded. So the roots of the characteristic polynomial of $\left(A-LC\right)$ are all in the left half plane at $-\omega_{o}$,

(10) $$\begin{array}{c} \lambda \left(s\right)=\left|sI-\left(A-LC\right)\right|={\left(s+\omega_{o}\right)}^{3} \end{array}$$

Therefore, *L* can be designed as

(11) $$\begin{array}{c} L=\left[\begin{array}{c} l_{1}\\ l_{2}\\ l_{3} \end{array}\right]=\left[\begin{array}{c} 3\omega_{o}-a_{1}\\ 3\omega_{o}^{2}-3a_{1}\omega_{o}-a_{0}+a_{1}^{2}\\ \omega_{o}^{3} \end{array}\right] \end{array}$$

In order for MCLESO to be BIBO stable, the observer bandwidth $\omega_{o}$ is the only tuning parameter.

Compared with LESO and MCLESO, the form of Equation (9) is the same, except that the matrix parameters are different. So some convergence analysis of MCLESO can be used for reference. The proof of these theories in LESO has been given in References [27,28]. For MCLESO, when the uncertainty $f^{\prime }$ or its derivative $\dot{f}{}^{\prime }$ is bounded, the estimation error is bounded. The upper bound of the estimation error monotonously decreases with the observer bandwidth. If the $f^{\prime }$ is globally Lipschitz with respect to *x*, the dynamic system describing the estimation error is asymptotically stable.

### 3.3. Dynamic Construction of the Model

In the case that MCLESO estimates the uncertain disturbance $f^{\prime }$ accurately in a certain frequency range, the extended state $z_{3}$ is compensated as shown in Figure 3. Let the control signal be

(12) [u=u0−z3u0−z3bb≈u0−fx+fwu0−fx+fwbb

Then taking Equation (12) into Equation (2),the controlled plant with *f* is constructed to the object form with $f_{0}$.

(13) θ¨=f0+fx+fw+bu≈f0+fx+fw+bu0−fx+fwu0−fx+fwbb=f0+bu0

And $f_{0}=-a_{1}\dot{\theta }-a_{0}\theta$ are available model dynamics. After the undesired disturbance is removed, the system is

(14) $$\begin{array}{c} \ddot{\theta }=-a_{1}\dot{\theta }-a_{0}\theta +bu_{0} \end{array}$$

Rewrite Equation (14) as transfer function,

(15) $$\begin{array}{c} G_{0}\left(s\right)=\frac{\theta (s)}{U_{0}\left(s\right)}=\frac{b}{s^{2}+a_{1}s+a_{0}} \end{array}$$

which is the available model by original identification.

When changing the load of the experimental platform, the gain decreases and resonance frequency shifts back by the actual measurement. The changes of the parameters are as follows.

(16) $$\begin{array}{c} \begin{array}{cc} K: & 2.6\to 1.6\\ \omega_{n}: & 6.5\;\mathrm{Hz}\to 5\;\mathrm{Hz} \end{array} \end{array}$$

**Figure 4:**
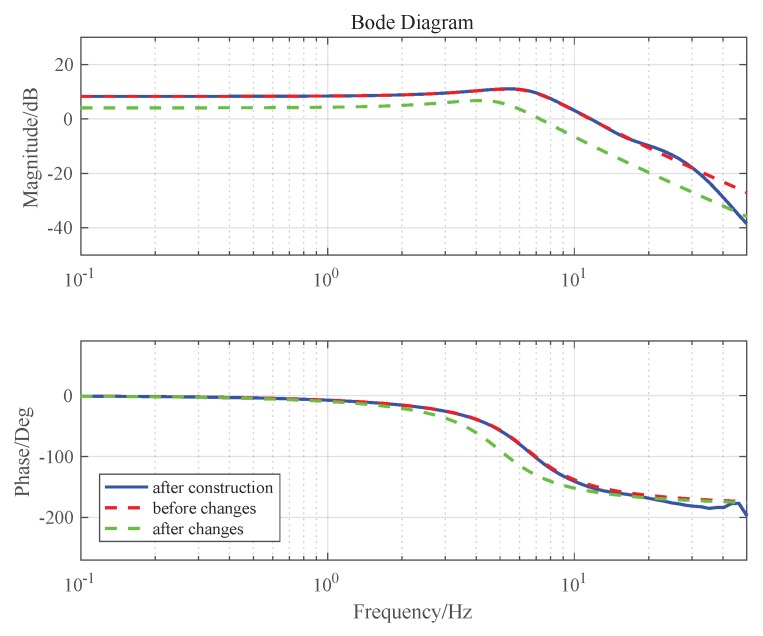
The Bode responses of the plant in simulation.

The MCLESO and the parameter *b* are designed based on a plant before the changes and constructs the changed plant. Bode responses of the plant after construction in simulation are shown in Figure 4.

**Figure 5:**
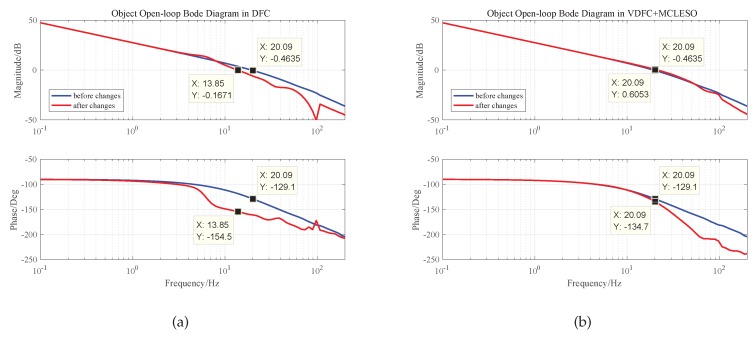
System open-loop Bode diagram before and after changes: (**a**) in DFC. (**b**) in VDFC with MCLESO.

We can see that the gain has changed by 38% in low frequency and the resonance frequency changes greatly. If the original controller is used regardless of the changes, the stability margin is greatly affected and the system is unstable. After dynamic construction, the internal changes are compensated and eliminated in 30 Hz. The changes of shear frequency and stability margin are still within acceptable limits. Set $\omega_{o}=200\;\mathrm{Hz}$ and the results are shown in Figure 5. The controllers of DFC and VDFC are the same, and the specific design is shown in Section 4.

## 4. Controller Design

### 4.1. Traditional DFC

**Figure 6:**
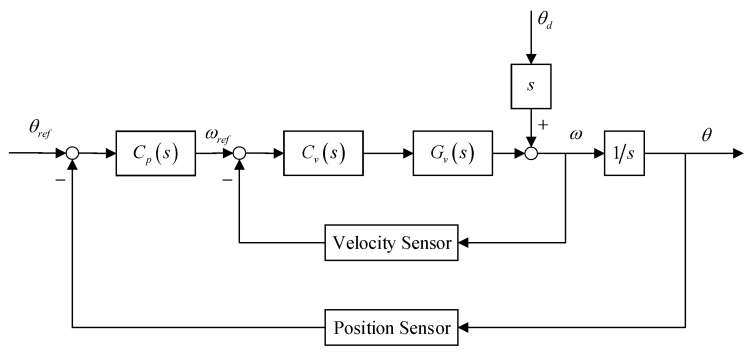
The block diagram of DFC.

The offset and jitter of the beam point calculated by position sensor and the velocity signal obtained by the velocity sensor are utilized to form a position-loop and velocity-loop in DFC, the block diagram of the traditional DFC is shown in Figure 6.

From Equation (1) and Figure 6, the velocity open-loop transfer function $G_{v}$ is as follows.

(17) $$\begin{array}{c} G_{v}\left(s\right)=\frac{\dot{\theta }\left(s\right)}{U(s)}=\frac{Ks}{\frac{s^{2}}{{\omega_{n}}^{2}}+\frac{2\zeta }{\omega_{n}}s+1} \end{array}$$

The ideal velocity controller $C_{v}$ is the inverse transfer function of $G_{v}$ with an integrator. The former is used to eliminate the zero-pole, the latter is to increase the system type and controller gain. In practice, a filter is added to avoid high frequency noise, where the design value of $T_{1}$ should be smaller than 0.01 to ensure that the bandwidth of control system is not too low.

The relative stability demand of the closed-loop system can be determined by the phase margin (PM) and magnitude margin (GM) of the open-loop system $G_{open}$, where $G_{open}=G_{v}\cdot C_{v}$,

(18) $$\begin{array}{c} GM>6dB,PM>\frac{\pi }{4} \end{array}$$

To satisfy Equation (18) by adjusting $K_{v}$, the velocity controller $C_{v}$ is as follows.

(19) $$\begin{array}{c} C_{v}\left(s\right)=K_{v}\cdot \frac{1}{s}\cdot \frac{\frac{s^{2}}{{\omega_{n}}^{2}}+\frac{2\zeta }{\omega_{n}}s+1}{s}\cdot \frac{1}{T_{1}s+1} \end{array}$$

Further, it is possible to fit the position open-loop transfer function when velocity closed-loop is completed

(20) $$\begin{array}{c} G_{p}\left(s\right)\approx \frac{K^{\prime }}{s\left(T_{e}s+1\right)} \end{array}$$

Moreover, the design method of the position controller $C_{p}$ is the same as the $C_{v}$, the existing integral part is used to increase gain, but the inverse of $G_{p}$ is not strictly a positive transfer function, so $T_{2}+1$ is employed.

(21) $$\begin{array}{c} G_{p}\left(s\right)\approx \frac{K^{\prime }}{s\left(T_{e}s+1\right)} \end{array}$$

Traditional DFC controllers are designed by the initial identification information of the controlled plant.

### 4.2. VDFC with MCLESO

**Figure 7:**
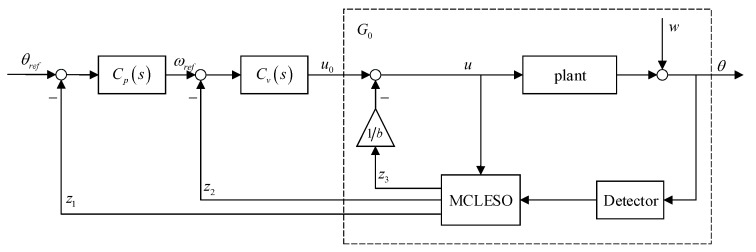
The block diagram of the VDFC with MCLESO.

The analysis in Section 3 shows that MCLESO can estimate and compensate the internal and external disturbance of the system. The changed plant is constructed to $G_{0}$ in a certain observation capability. This provides guidance for subsequent controller design. Meanwhile, the output and its derivative, the position and velocity signal, are observed by the ($z_{1},z_{2}$) of MCLESO. So, these estimated outputs of observer are composed to implement virtual DFC (VDFC). The block diagram of the VDFC with MCLESO is shown in Figure 7.

Most conveniently, benefiting from the model-construction after uncertain disturbance compensation, the velocity controller and position controller can use traditional DFC controllers without redesigning them.

### 4.3. Classical LADRC

Classical LADRC controller and observer are designed and tuned according to the Reference [25]. A common rule of thumb is to choose $\omega_{o}=3∼5\omega_{c}$, where $\omega_{c}$ is the controller bandwidth of LADRC. The common linear PD control law is

(22) $$\begin{array}{c} u_{0}=k_{p}\left(\theta_{ref}-z_{1}\right)-k_{d}z_{2} \end{array}$$

where, $k_{p}=\omega_{c}^{2},k_{d}=2\omega_{c}$. It is noted that the observer bandwidth of LESO is the same as that of MCLESO for comparison.

## 5. Experimental Verification

**Figure 8:**
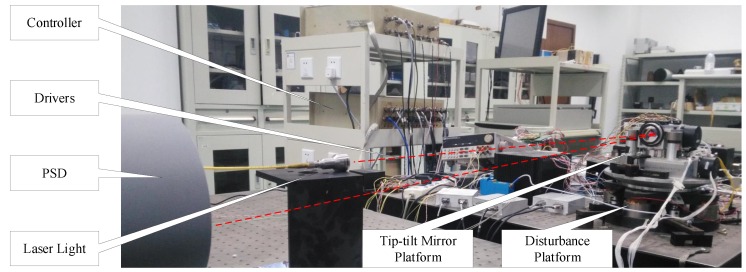
The experimental apparatus.

The tip-tilt mirror control system is a two-axis system. This experiment aims at one axis due to the symmetry of the two axes. As shown in Figure 8, the laser light is used to simulate the beacon of light. An apparatus constructed by two superimposed tip-tilt mirror platforms is used to verify the previous analysis. One is used to stabilize the light, the other is to simulate disturbance which is measured by position sensors. The tip-tilt mirror platform is mounted on the disturbance platform, and both are driven by the voice coil motors. The mirror reflects the laser light into the PSD, which detects the stabilization error at the sampling rate of 5 kHz.

The open-loop position transfer function of the tip-tilt mirror is measured by spectral analyzer, which is shown in Equation (23), where $K=2.6,\zeta =0.4,\omega_{n}=6.5\;\mathrm{Hz}$ are initial approximations.

(23) $$\begin{array}{c} G\left(s\right)=\frac{\theta (s)}{U(s)}=\frac{4336.7}{s^{2}+32.67s+1668} \end{array}$$

As shown in Equation (19), the velocity controller is

(24) $$\begin{array}{c} C_{v}\left(s\right)=\frac{150}{s^{2}}\cdot \frac{0.00059953s^{2}+0.0196s+1}{0.00053052s+1} \end{array}$$

where, the parameters are Kv=150,T1=112π×3002π×300.

The position controller is

(25) $$\begin{array}{c} C_{p}\left(s\right)=200\times \frac{0.00079577s+1}{0.00053052s+1} \end{array}$$

where, the parameters are Kp=200,T2=112π×3002π×300,Te=112π×2002π×200.

The observer bandwidth of MCLESO and classical LESO are

(26) $$\begin{array}{c} \omega_{o\_MCLESO}=\omega_{o\_LESO}=100\;\mathrm{Hz} \end{array}$$

to compare estimation and compensation capabilities.

### 5.1. Discrete Implementation of MCLESO

In the actual computer implementation process, the continuous observer algorithm needs to be discretized. The computer runs at 5 kHz in the experiment, and the discrete method uses the current zero order hold (ZOH) method. The estimation accuracy and stability of ZOH and first order hold (FOH) method are better than Euler, and the ZOH is superior to the FOH in reducing the delay associated with the sampling process without additional complexity to the user, which is shown in [31].

### 5.2. Comparison Results of DSA

**Figure 9:**
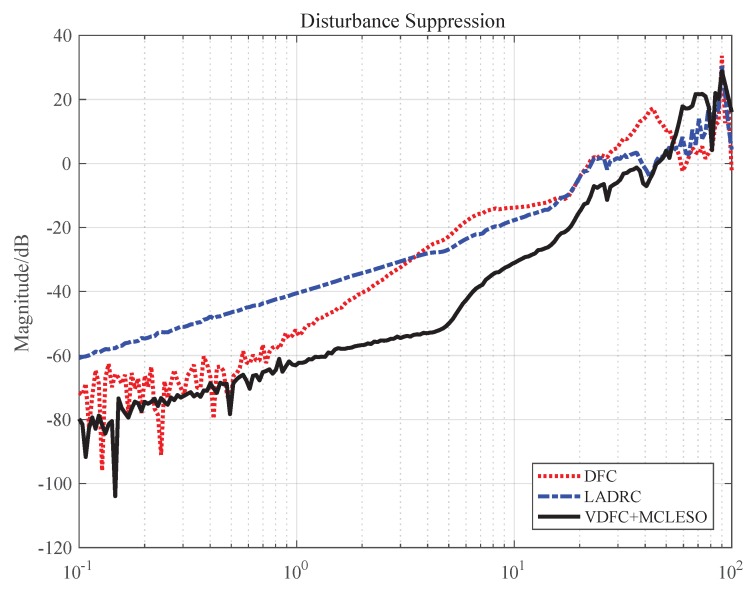
Disturbance suppression ability of traditional DFC, classical LADRC and VDFC with MCLESO.

The traditional DFC, the classical LADRC and the VDFC with MCLESO were applied on the tip-tilt mirror platform when the disturbance platform works. The disturbance input of the mirror platform is the value measured by the sensors on the disturbance platform. So, the DSA is defined as the frequency characteristics from the final stabilization error to the disturbance input. The comparison results of DSA of three methods in the range from 0.1 to 100 Hz are shown in Figure 9.

### 5.3. Discussion

As shown in Figure 9, it is obvious that the DSA of VDFC with MCLESO is stronger than LADRC and DFC in the low and medium frequency, which verifies the previous analysis. Compared with the traditional DFC, because of the observation and compensation of the disturbance by adding MCLESO, the undesired impact including the unknown internal dynamics and external disturbance is eliminated. The stability margin changes with the same controllers which have been analyzed in the previous simulations. The DSA is also greatly enhanced in the range from 1 to 30 Hz. The maximum improved effect performances a 30 dB’s advantage at 5 Hz. At the same time, the proposed strategy can directly use the traditional DFC controller and save extra space and cost of the velocity sensors. Compared with the classical LESO, MCLESO can better estimate and compensate disturbance in the same observer bandwidth. In theory, the higher the bandwidth, the smaller the observation error, but the excessive bandwidth will introduce high-frequency noise and the saturation of the feedback gain, so it is necessary to think about using ESO to enhance DSA with limited bandwidth. It is noted that, compared with LESO of adding model information, MCLESO changes the compensation method, and the available dynamics continue to remain in the original dynamics of the system.

## 6. Conclusions

In this paper, a VDFC with MCLESO which is applied in the fast tip-tilt mirror platform to improve the DSA of the fine tracking system in free space optical communication. The implementation of the proposed control structure, the construction effect of MCLESO using the available model information, and the design of the controllers are provided in detail. Since the parameters of the model cannot be accurately identified and easily changed, the MCLESO can construct known model dynamics by observing and compensating the unknown internal and external disturbance. The controllers of the DFC system are, therefore, employed directly in the VDFC. Moreover, MCLESO improves the estimation accuracy of the total disturbance compared to classical LESO without increasing the observer bandwidth. The proposed VDFC with MCLESO enhances the DSA of the tip-tilt mirror in low and medium frequency, and ensures that the stable alignment and transmission of the optical communication are better linked. Meanwhile, this method saves cost and space of a fast tip-tilt mirror platform, which is beneficial to its miniaturization and rapid deflection.

In the future, our subsequent work will focus on theoretical analysis of the stability performance of the observer in the frequency domain, such as the range of the controlled object parameters, the performance limit and suppression of nonlinear disturbances.

## References

[1] Rabinovich W., Moore C., Mahon R., Goetz P., Burris H., Ferraro M., Murphy J., Thomas L., Gilbreath G., Vilcheck M. (2015). Free-space optical communications research and demonstrations at the US Naval Research Laboratory. Appl. Opt..

[2] Toyoshima M., Fuse T., Kolev D.R., Takenaka H., Munemasa Y., Iwakiri N., Suzuki K., Koyama Y., Kubooka T., Akioka M. Current status of research and development on space laser communications technologies and future plans in NICT. Proceedings of the 2015 IEEE International Conference on Space Optical Systems and Applications (ICSOS).

[3] Shao B., Sun L., Qu D., Wang J., Qin C. (2006). Design of fine pointing tip/tilt mirror of ATP system for free space optical communication. Opt. Precis. Eng..

[4] Ortiz G.G., Lee S., Monacos S.P., Wright M.W., Biswas A. Design and development of a robust ATP subsystem for the altair UAV-to-ground lasercomm 2.5-Gbps demonstration. Proceedings of the Free-Space Laser Communication Technologies XV, International Society for Optics and Photonics.

[5] Ma J., Tang T. (2013). Review of compound axis servomechanism tracking control technology. Infrared Laser Eng..

[6] Peng X., Ma J. (1994). The Research and Experiments of Compound-Axis Control System in Opto-Electronic Accurate Tracking. Opto-Electronic Eng..

[7] Kulcsár C., Sivo G., Raynaud H.F., Neichel B., Rigaut F., Christou J., Guesalaga A., Correia C., Véran J.P., Gendron E. Vibrations in AO control: A short analysis of on-sky data around the world. Proceedings of the Adaptive Optics Systems III International Society for Optics and Photonics.

[8] Dong R., Ai Y., Xiao Y., Shan X. (2012). Design and communication experiment of fine tracking system for free space optic. Infrared Laser Eng..

[9] Corke P.I., Hutchinson S.A. (2001). A new partitioned approach to image-based visual servo control. IEEE Trans. Robot. Autom..

[10] Gilmore J.P., Luniewicz M.F., Sargent D. Enhanced precision pointing jitter suppression system. Proceedings of the SPIE 4632, Laser and Beam Control Technologies.

[11] Yahalom R., Moslehi B., Oblea L., Sotoudeh V., Ha J. Low-cost, compact fiber-optic gyroscope for super-stable line-of-sight stabilization. Proceedings of the IEEE/ION Position, Location and Navigation Symposium.

[12] Tian J., Yang W., Peng Z., Tang T., Li Z. (2016). Application of MEMS accelerometers and gyroscopes in fast steering mirror control systems. Sensors.

[13] Yasuda M., Osaka T., Ikeda M. Feedforward control of a vibration isolation system for disturbance suppression. Proceedings of the 35th IEEE Conference on Decision and Control.

[14] Glück M., Pott J.U., Sawodny O. (2016). Piezo-actuated vibration disturbance mirror for investigating accelerometer-based tip-tilt reconstruction in large telescopes. IFAC-PapersOnLine.

[15] Ohnishi K., Shibata M., Murakami T. (1996). Motion control for advanced mechatronics. Ieee/Asme Trans. Mech..

[16] Sariyildiz E., Ohnishi K. (2014). Stability and robustness of disturbance-observer-based motion control systems. IEEE Trans. Ind. Electron..

[17] Tang T., Niu S., Chen X., Qi B. (2018). Disturbance Observer-Based Control of Tip-Tilt Mirror for Mitigating Telescope Vibrations. IEEE Trans. Instrum. Meas..

[18] Chen W., Yang J., Guo L., Li S. (2015). Disturbance-observer-based control and related methods—An overview. IEEE Trans. Ind. Electron..

[19] Panchade V., Chile R., Patre B. (2013). A survey on sliding mode control strategies for induction motors. Annu. Rev. Control..

[20] Ren Y., Wang W., Wang Y. (2018). Incremental H∞ control for switched nonlinear systems. Appl. Math. Comput..

[21] Li J., Xia Y., Qi X., Zhao P. (2017). Robust absolute stability analysis for interval nonlinear active disturbance rejection based control system. ISA Trans..

[22] Han J. (1989). Control theory, is it a model analysis approach or a direct control approach?. J. Syst. Sci. Math. Sci..

[23] Han J. (2009). From PID to Active Disturbance Rejection Control. IEEE Trans. Ind. Electron..

[24] Gao Z. Active disturbance rejection control: A paradigm shift in feedback control system design. Proceedings of the 2006 American Control Conference.

[25] Gao Z. Scaling and bandwidth-parameterization based controller tuning. Proceedings of the 2003 American Control Conference.

[26] Tian G., Gao Z. Frequency response analysis of active disturbance rejection based control system. Proceedings of the 2007 IEEE International Conference on Control Applications.

[27] Zheng Q., Gao L.Q., Gao Z. (2012). On validation of extended state observer through analysis and experimentation. J. Dyn. Syst. Meas. Control..

[28] Yang X., Huang Y. Capabilities of extended state observer for estimating uncertainties. Proceedings of the 2009 American Control Conference.

[29] Sun L., Li D., Gao Z., Yang Z., Zhao S. (2016). Combined feedforward and model-assisted active disturbance rejection control for non-minimum phase system. ISA Trans..

[30] Shao X., Liu N., Liu J., Wang H. (2018). Model-assisted extended state observer and dynamic surface control–based trajectory tracking for quadrotors via output-feedback mechanism. Int. J. Robust Nonlinear Control..

[31] Miklosovic R., Radke A., Gao Z. Discrete implementation and generalization of the extended state observer. Proceedings of the 2006 American Control Conference.

